# High-quality metagenome-assembled genome sequences of *Bacteroidota* and *Pseudomonadota* bacteria, assembled from a manganese(II)-oxidizing biofilm reactor

**DOI:** 10.1128/mra.00588-26

**Published:** 2026-07-10

**Authors:** Masataka Aoki, Naoki Wakui, Kazuyuki Hayashi, Kazuaki Syutsubo

**Affiliations:** 1 Regional Environment Conservation Division, National Institute for Environmental Studies 13585, Tsukuba, Ibaraki, Japan; 2 Department of Electrical and Electronic Systems Engineering, National Institute of Technology, Nagaoka College 118102, Nagaoka, Niigata, Japan; 3 Department of Civil Engineering, National Institute of Technology, Wakayama College, Gobo, Wakayama, Japan; 4 Research Center for Water Environment Technology, School of Engineering, The University of Tokyo 13143, Bunkyo-Ku, Tokyo, Japan; California State University San Marcos, San Marcos, California, USA

**Keywords:** Mn(II) oxidation, *Pseudomonadota*, *Bacteroidota*, metagenome-assembled genome

## Abstract

We report five high-quality, potentially novel metagenome-assembled genomes (MAGs) recovered from a manganese(II)-oxidizing biofilm reactor. Affiliated with *Bacteroidota* and *Pseudomonadota*, these MAGs provide a genomic basis for understanding the ecology and metabolic potential of Mn(II)-oxidizing systems and represent a valuable resource for future functional studies of biofilm-mediated metal cycling.

## ANNOUNCEMENT

Manganese(II)-oxidizing biofilms play a critical role in geochemical cycling and metal recovery through manganese oxide (MnOx) production (1). Despite advances in the engineering of such systems, genomic insights remain limited. Previously, we demonstrated successful Mn(II) oxidation in a laboratory-scale biofilm reactor using aspen wood and polyurethane sponge media at the National Institute for Environmental Studies, Japan (36°3′1″ N, 140°7′9″ E) (2). The system was supplied with synthetic freshwater containing 5 mg L^−1^ Mn(II). Here, we report high-quality metagenome-assembled genomes (MAGs) recovered from this reactor.

At the end of reactor operation (day 469), biofilms for metagenomic sequencing were collected from polyurethane sponges at the bottom of the reactor, where MnOx was present. Details of reactor configuration and operation, biofilm recovery, and MnOx detection have been described previously (2). DNA was extracted from biofilms using the Extrap Soil DNA Kit Plus Ver.2 (BioDynamics Laboratory Inc., Japan), with cell disruption performed at 6.0 m s^−1^ for 45 s using a FastPrep-24 5G instrument (MP Biomedicals, USA) (2). The extracted DNA was eluted in 100 µL Tris-EDTA buffer (pH 8.0). Library preparation was performed using the MGIEasy Fast FS Library Prep Set V2.0 (MGI Tech, China). Circular DNA was generated using the prepared library and the MGIEasy Dual Barcode Circularization Kit (MGI Tech). DNA nanoballs were subsequently produced using the DNBSEQ-G400RS High-throughput Sequencing Kit and the High-throughput Paired-End Sequencing Primer Kit (App-D; MGI Tech). Paired-end sequencing (2 × 150 bp) was performed using a DNBSEQ-G400 sequencer (MGI Tech), generating 41,777,092 reads (12,533,127,600 bp).

Reads were quality-filtered and adapter-trimmed using bbduk.sh from BBMap v39.01 (parameters: ktrim = r ref = adapters k = 23 mink = 11 hdist = 1 tpe tbo qtrim = r trimq = 10 minlength = 40 maxns = 1 minavgquality = 15) (https://sourceforge.net/projects/bbmap/). Human-derived sequences were removed by mapping reads to a human reference genome (https://zenodo.org/record/1208052#.X1hBFWf7SdY) using bbmap.sh (parameters: quickmatch fast untrim minid = 0.95 maxindel = 3 bwr = 0.16 bw = 12 minhits = 2 qtrim = rl trimq = 10). Quality-filtered reads were assembled using SPAdes v3.15.5 (parameters: --meta --disable-gzip-output –k 21,33,55,77,99,127) (3). Short contigs (<200 bp) were removed using SeqKit v2.6.1 (4). Remaining contigs were binned using MaxBin2 v2.2.7 (5) and MetaBAT 2 v2.15 (6). The resulting MAGs were refined using MAGScoT v1.0.0 (parameters: -b 1 -c 1 -m 1) (7). MAGs consisting of <100 contigs were annotated using DFAST v1.3.6 (8). MAG quality was assessed using CheckM2 v1.1.0 (9). Mean genome coverage was calculated using CoverM v0.7.0 (parameters: genome -m mean) (10). Genome and 16S ribosomal RNA taxonomies were assigned using GTDB-Tk v2.6.1 with the R226 database (11) and mothur v1.48.4 using the “classify.seqs” command (12) with the SILVA 138.2 database (13), respectively. Closest isolates were identified using the EzBioCloud 16S-based ID service (database v2025.04.21) (14). Putative Mn(II)-oxidizing genes were identified using the MnOxGeneTool-hmm module of MnOxGeneTool v1.0.0 (15). Default parameters were used, unless otherwise specified.

Table 1 summarizes the genome statistics and taxonomic classifications of the five *Bacteroidota* and *Pseudomonadota* MAGs, all of which meet high-quality MAG criteria (16). Genome taxonomy indicated that these MAGs likely represent novel taxa. Notably, four MAGs contained putative Mn(II)-oxidizing genes, supporting their potential direct involvement in biofilm-mediated Mn transformation.

**TABLE 1:** Genomic and taxonomic information of five high-quality MAGs assembled from the manganese(II)-oxidizing biofilm reactor^*a*^^,*d*^

MAG	WMNR-BS1	WMNR-BS2	WMNR-BS4	WMNR-BS6	WMNR-BS7
MAG size (bp)	3,373,803	5,915,308	5,818,112	4,264,392	2,933,178
No. of contigs	60	9	73	3	18
GC content (%)	39.2	65.8	67.5	52.5	49.3
*N*_50_ length (bp)	95,793	1,439,761	137,528	3,243,235	231,321
Mean coverage (×)	45	83	31	34	15
No. of CDS	2,997	5,691	5,585	3,631	2,474
No. of rRNA genes (No. of 5S, 16S, and 23S)	3 (1, 1, 1)	3 (1, 1, 1)	3 (1, 1, 1)	4 (1, 2, 1)	3 (1, 1, 1)
No. of tRNA genes (No. of tRNA types)	39 (20)	49 (21)	53 (20)	44 (20)	36 (20)
Completeness (%)	99.24	99.83	97.20	99.32	98.32
Contamination (%)	0.07	0.92	1.47	0.28	0.18
GTDB taxonomy					
Phylum	*Bacteroidota*	*Pseudomonadota*	*Pseudomonadota*	*Bacteroidota*	*Bacteroidota*
Family	*Chitinophagaceae*	SG8-39	Bin65	*Cyclobacteriaceae*	UKL13-3
Genus	*Sediminibacterium*	SZAS-79	SBBV01	ELB16-189	JAJTFA01
Species	–^*b*^	–	–	–	–
16S rRNA taxonomy					
Phylum	*Bacteroidota*	*Pseudomonadota*	*Pseudomonadota*	*Bacteroidota*	*Bacteroidota*
Family	*Chitinophagaceae*	*Nitrosomonadaceae*	*Alphaproteobacteria incertae sedis* family	*Microscillaceae*	NS11-12 marine group
Genus	*Sediminibacterium*	MND1	*Alphaproteobacteria incertae sedis* genus	*Chryseotalea*	NS11-12 marine group *incertae sedis* genus
Closest isolate with validated name (16S rRNA gene similarity [%]; accession no.)	*Sediminibacterium goheungense* DSM 28323^T^ (99.59; jgi.1108054)	*Noviherbaspirillum aridicola* NCCP-691^T^ (91.90; LC193938)	*Hypericibacter terrae* R5913^T^ (90.64; MG271952)	*Chryseotalea sanaruensis* Ys^T^ (89.62; LC349727)	*Solitalea koreensis* R2A36-4^T^ (86.38; EU787448)
Putative Mn(II)-oxidizing gene (locus tag)^*c*^	*katG* (WMNRBS1_16660)	*katG* (WMNRBS2_31890)	*moxA* (WMNRBS4_04030) *katG* (WMNRBS4_44650)	*katG* (WMNRBS6_01460)	Not detected

^*a*^ High-quality MAG is defined as one with completeness >90%, contamination <5%, presence of the 5S, 16S, and 23S rRNA genes, and tRNA genes for at least 18 amino acids (16).

^*b*^ –, the MAG sequence is potentially novel.

^*c*^ Identified using the MnOxGeneTool-hmm module of MnOxGeneTool v1.0.0 (15).

^*d*^ CDS, coding sequence; GTDB, Genome Taxonomy Database; MAG, metagenome-assembled genome; rRNA, ribosomal RNA; tRNA, transfer RNA.

## Data Availability

The annotated WMNR-BS1, WMNR-BS2, WMNR-BS4, WMNR-BS6, and WMNR-BS7 MAGs are available in DDBJ/ENA/GenBank under accession numbers BAAKGO000000000, BAAKGP000000000, BAAKGQ000000000, BAAKGR000000000, and BAAKGS000000000, respectively. The raw DNBSeq read data have been deposited in the Sequence Read Archive under accession number DRR916421.
